# Prevalence and Predictors of Sexual Difficulties and Associated Distress Among Partnered, Sexually Active Older Women in Norway, Denmark, Belgium, and Portugal

**DOI:** 10.1007/s10508-020-01742-7

**Published:** 2020-06-12

**Authors:** Cynthia A. Graham, Aleksandar Štulhofer, Theis Lange, Gert Martin Hald, Ana A. Carvalheira, Paul Enzlin, Bente Træen

**Affiliations:** 1 Department of Psychology, Faculty of Environmental and Life Sciences, University of Southampton, Shackleton Building (B44), Room 44/3073, Southampton, SO17 1BJ UK; 2 Department of Sociology, University of Zagreb, Zagreb, Croatia; 3 Section of Biostatistics, University of Copenhagen, Copenhagen, Denmark; 4 Department of Public Health, University of Copenhagen, Copenhagen, Denmark; 5 Department of Psychology, University of Lisbon, Lisbon, Portugal; 6 Department of Neurosciences, KU Leuven, Institute for Family and Sexuality Studies, Leuven, Belgium; 7 Department of Psychology, University of Oslo, Oslo, Norway

**Keywords:** Sexual problems, Sexual function, Sexual distress, Cross-cultural, Older women, DSM-5

## Abstract

There has been little comparative, cross-cultural research on sexual difficulties and associated distress, and factors associated with these, among older women. Therefore, the aim of this study was to investigate prevalence rates of sexual difficulties, distress related to these difficulties, and associated sociodemographic, relational, and health factors, among sexually active older women (60–75 years) in committed relationships across four European countries (Norway, Denmark, Belgium, and Portugal). These data could inform us about what differentiates women who do and do not experience distressing sexual difficulties and facilitate the identification of older women who might benefit from clinical interventions as well as the development of new interventions. In total, 1057 women (357 Norwegian; 322 Danish; 237 Belgian; 141 Portuguese) completed a cross-sectional questionnaire assessing six sexual difficulties—vaginal dryness, orgasmic difficulties, lacking interest in sex, lacking enjoyment in sex, pain during sex, and no excitement/arousal during sex—and associated distress. We found a high prevalence of sexual difficulties lasting 3 months or longer in the past year (between 23.5 and 50.2%, depending on the specific difficulty). With the exception of vaginal dryness and pain during sex, however, the majority of women reporting sexual difficulties (50.0% to 86.1%, depending on the specific difficulty) reported no or mild distress. There were relatively few cross-country differences, either in the prevalence of sexual difficulties or related distress. Few sociodemographic or health variables were associated with distressing sexual difficulties, but higher sexual intimacy, higher emotional intimacy, and better mental health were associated with less distress about some sexual difficulties. The findings underline the importance of healthcare professionals asking older women about sexual function and especially associated distress, and suggest that careful attention to the psychological and relationship context of these sexual difficulties is needed, as these could be important targets in the treatment process.

## Introduction

Research suggests that while sexual difficulties are more prevalent among older women compared to younger women (Hayes & Dennerstein, 2005; Hendrickx, Gijs, & Enzlin, 2015; Mitchell et al., 2013; Peixoto & Nobre, 2015), older populations of women tend to report less distress about sexual difficulties (Bancroft, Loftus, & Long, 2003; Lee, Nazroo, O’Connor, Blake, & Pendleton, 2016; Santos-Iglesias, Byers, & Moglia, 2016). In the English Longitudinal Study of Ageing, with increasing age, women reported *less* concern about sexual problems and lower levels of concern compared to men (Lee et al., 2016). Among sexually active women aged 65–74 years in the third wave of the British National Surveys of Sexual Attitudes and Lifestyles (Natsal-3), 55.7% reported one or more sexual problems lasting 3 months or longer in the last year (Mitchell et al., 2016). The most common problems were lacking interest in sex, “uncomfortably dry vagina,” and difficulty reaching orgasm. However, only 9.5% of women in this age group reported “distress” or “worry” about their sex life (compared with 12.7% of women aged 45–54 years). In a further analysis of Natsal-3 data, estimates of the prevalence of sexual function problems meeting *Diagnostic and Statistical Manual of Mental Disorders* (DSM-5) (American Psychiatric Association, 2013) morbidity criteria (“fairly” or “very” distressed, duration of problem at least 6 months, and symptoms occur “very often” or “always”) were obtained (Mitchell et al., 2016). For women aged 65–74 years, less than two percent met all of the above morbidity criteria, compared to 2.8% to 5.3% in the younger age groups.

Although most surveys in recent years have assessed distress about sexual difficulties in addition to sexual difficulty symptoms, we know surprisingly little about what differentiates women (of any age group) who report distress about a sexual difficulty from those who do not. Most studies to date have focused on possible psychosocial, sociodemographic, and relationship predictors of the occurrence of sexual difficulties and not on predictors of associated distress; this literature is reviewed below.

### Sociodemographic Factors Associated with Sexual Problems and Associated Distress

Research on the role of sociodemographic factors such as age, education, and religiosity as predictors of sexual problems among older adult women is limited (Træen et al., 2016). There are inconsistent findings on the associations of age with sexual problems in women of mixed ages (for review, see Træen et al., 2016).

There may also be differential associations between age and specific sexual difficulties. For example, most studies have reported a positive relationship between age and lubrication difficulties (Laumann, Das, & Waite, 2008; Peixoto & Nobre, 2015). However, in the Global Survey of Sexual Attitudes and Lifestyles, women aged 70–80 years were no more likely to report lubrication problems than women aged 40–49 (Laumann et al., 2006). Pain during sex, on the other hand, tends to be more common among younger than older women (Mitchell et al., 2017). In one of the few studies on predictors of sexual distress, Hendrickx et al. (2015) sampled 15,048 heterosexual women and found that overall sexual distress was more common among younger women than older women. The prevalence of two sexual problems—orgasm difficulties and painful sex—showed a U-shaped association with age, being lowest in women aged 40–49 years old, and higher in both younger and older women.

Evidence on the associations between educational background and sexual difficulties has also been mixed. Several studies of mixed-age groups reported that women with higher levels of education were less likely to report sexual problems (Abdo, Oliveira, Moreira, & Fittipaldi, 2004; Çayan et al., 2004; Peixoto & Nobre, 2015; Shifren, Monz, Russo, Segreti, & Johannes, 2008), although some studies have found the opposite (Christensen et al., 2011).

Few studies have assessed associations between religiosity and sexual difficulties, but levels of religiosity have been associated with sexual behavior in older adults. In the U.S. National Health and Social Life Survey, religious older adults were less likely to think about sex and less likely to engage in varied sexual activities such as oral sex or anal sex, masturbation, or have multiple partners (Laumann, Gagnon, Michaels, & Michaels, 1994). In a more recent, nationally representative survey of older adults, stronger religious influence was associated with higher physical and emotional satisfaction with sex, but only for men (Iveniuk, O’Muircheartaigh, & Cagney, 2016). Studies on possible associations between religiosity and sexual difficulties and associated distress among older adults are lacking.

### Psychosocial and Relationship Factors Associated with Sexual Problems and Associated Distress

#### Emotional and Sexual Intimacy

It has been suggested that emotional and sexual intimacy should be seen as two different constructs and that both may be particularly important predictors of sexual problems and associated distress among older populations of women (Štulhofer, Jurin, Graham, Enzlin, & Træen, 2019). While emotional intimacy refers to the overall quality of closeness an individual has with their partner (Sinclair & Dowdy, 2005), sexual intimacy is the degree of closeness an individual has with their partner when they have sex together. Indeed, among samples of women of varied ages, emotional closeness with a partner during sexual activity has been linked to less distress about sex. In a national survey of American women aged 20–65 years, one of the strongest predictors of distress about their sexual relationships was a measure of the “emotional relationship” with partners during sexual activity (Bancroft et al., 2003). Furthermore, older individuals consider a wider range of sexual behaviors as sexual activity (Hinchliff & Gott, 2004), and there is evidence that emotional and physical intimacy become increasingly important as people age (Fileborn et al., 2017; Freak-Poli et al., 2017; Heiman et al., 2011; Laumann et al., 2006). In a longitudinal population-based study of older German women and men that assessed individuals at ages 63, 67, and 74 years, both men and women at age 74 rated “affection” as more important than sexual activity (Müller, Nienaber, Reis, Kropp, & Meyer, 2014). Several qualitative studies have also highlighted the importance of emotional closeness and intimacy during sexual activity for older adults (Fileborn et al., 2017; Ménard et al., 2015).

#### Mental and Physical Health

Sexual problems in older adults are associated with poorer physical health (DeLamater, 2012; Field et al., 2013), and there is some evidence that physical health problems may have greater negative effects on sexual function for women than for men (Erens et al., 2019; Laumann et al., 2008). Fewer studies have focused on the association between mental health and sexual problems in older adults. In one population-based U.S. study of older adults aged 50–99, all of whom had a partner, depressive symptoms were negatively associated with all aspects of sexual health, even after adjusting for age and physical health (Wang et al., 2015). In this study, Wang et al. (2015) defined sexual health broadly as including “problems with the relationship, discussing sex, frequency of activity, satisfaction, desire, rejection of sexual overtures, and dysfunction” (p. 229).

#### Cross-cultural Aspects

There has been very little comparative research on sexual difficulties and associated distress among older populations (Heiman et al., 2011; Laumann et al., 2006). This is surprising given that gender roles and attitudes toward sex show considerable cross-cultural variation. Few studies have examined associations between these attitudes and the prevalence of sexual problems and associated distress. In the UK Natsal-3 study, male and female respondents across all ages who endorsed the statement “people want less sex as they age” were more likely to report lack of interest in sex (Graham et al., 2017). Similarly, women who endorsed the view that “men have a higher sex drive than women” were also significantly more likely to report lack of interest in sex (the opposite relationship was found for men).

Some research has pointed to a possible North–South European difference in sexual behavior (Træen, Štulhofer, & Landripet, 2011) and attitudes (Træen, Carvalheira, Hald, Lange, & Kvalem, 2019a). Sexual cultures of Northern European countries tend to be characterized as more liberal and more accepting of sexual equality across gender and age groups (Haavio-Mannila & Kontula, 2003). In contrast, individuals in Southern European countries tend to adhere more strongly to traditional male and female sexual roles that hold women should be more passive and submissive (Štulhofer, Šoh, Jelaska, Baćak, & Landripet, 2011). One possible explanation for cross-country differences in the prevalence of sexual difficulties might be related to these differences in gender roles.

#### The Current Study

The framework of “successful aging” that recognizes the importance of sexuality for older men and women guided the design of the current study. The important role of sexuality in successful aging has increasingly been recognized (Štulhofer, Hinchliff, Jurin, Hald, & Træen, 2018). Successful aging has been significantly associated with sexual satisfaction (Woloski-Wruble, Oliel, Leefsma, & Hochner-Celnikier, 2010), and with sexual desire and activity (Thompson et al., 2011), even in the context where there are important age-related changes in sexual frequency and function. In this study, we focused on sexual problems and associated distress as two possible threats of successful aging and sought to address two gaps in the literature on sexuality among older women: the lack of understanding about what predicts reported distress about specific sexual difficulties, and the lack of cross-cultural comparative data on sexual difficulties and associated distress. Thus, the aim of the current study was to investigate prevalence rates of sexual difficulties, distressing sexual difficulties, and factors associated with these, among sexually active older women (aged 60–75 years) in a committed relationship in four European countries (Norway, Denmark, Belgium, and Portugal). We believe that a better understanding of what differentiates women who do and do not experience distressing sexual difficulties will facilitate the identification of older women who might benefit from clinical interventions and inform the development of new interventions. Because of the dearth of previous evidence on these topics among older adults, no specific hypotheses were formulated. The specific research questions (RQs) were:

RQ1: What are the prevalence rates of sexual difficulties and distressing sexual difficulties in older women in Norway, Denmark, Belgium, and Portugal?

RQ2: Do relevant sociodemographic factors (age, education, and religiosity) predict distress about sexual difficulties in older women?

RQ3: Are relational (emotional and sexual intimacy), and health (mental and physical health) factors associated with distress about specific sexual difficulties in older women?

## Method

### Participants and Procedure

Survey data were collected in national probability-based samples of women aged 60–75 years in Norway, Denmark, Belgium, and Portugal between October 2016 and January 2017. Data collection was conducted by the marketing company IPSOS in cooperation with researchers at the University of Oslo.

Initial recruitment interviews were done by telephone to obtain nationally representative samples of the population aged 60–75 in each country. Only data obtained from women are presented here; findings related to men are reported elsewhere (Hald et al., 2019). For this analytic sample, inclusion criteria were: women aged 60–75 years; who were in a steady committed relationship (68.5% of the total sample); and who were sexually active during the past 12 months (71.7% of the total sample).

The questionnaire was first developed in English and then translated—using forward and backward translation procedures—into local languages by the principal investigators and by IPSOS staff in each country. When translation was complete, telephone recruitment of participants began. With the exception of Portugal, where a comprehensive telephone register does not exist, national phone registries were used to obtain lists of possible respondents. IPSOS used a common procedure when recruiting respondents for surveys in Portugal: (1) telephone numbers were first randomly selected from fixed phone directories and IPSOS’s own database of phone numbers; (2) to obtain a distribution representative of the population, participants were selected by age and gender; and (3) due to illiteracy problems, participants who had not completed primary school (ISCED 235 1) were excluded from the sample. For all countries, telephone recruitment was conducted from October to December 2016.

The procedure was as follows: First, the participants were contacted by IPSOS by phone. If they agreed to participate, they answered a short background questionnaire. IPSOS had the names and addresses of participants registered, and a questionnaire was sent to them the following day. This questionnaire was coded, so reminders were only sent to those who did not respond. We as researchers received an anonymized SPSS file that could not be traced back to single individuals. To preserve participants’ anonymity, we did not require written consent.

Women who agreed to participate received a postal, anonymous, self-administered questionnaire, including a Freepost envelope to return the completed questionnaire. Two reminders were sent, the first 1 week after the questionnaire was received by the participant, and the second 1 week later. After a discussion with IPSOS in Portugal, it was decided to deliver the reminders by phone there. Unfortunately, 502 potential participants in Portugal could not be reached. Of the 1498 Portuguese individuals contacted by phone, 561 declined participation after having received the questionnaire.

All participants who returned completed questionnaires received a small gift from IPSOS for their efforts. Overall response rates, i.e., the proportion of women who agreed to participate and completed the questionnaire, were 68% in Norway, 52% in Denmark, 57% in Belgium, and 26% in Portugal. In total, 1057 women participated (357 Norwegian; 322 Danish; 237 Belgian; 141 Portuguese). A more detailed description of the sample and the procedure is given elsewhere (Træen et al., 2019b).

### Measures

Age was indicated by year of birth. Relationship status was assessed with the question “Do you currently have a steady/committed relationship with anybody? A steady/committed relationship also includes married/cohabiting persons.” Response options were 1 = Yes, 2 = No, and 3 = Unsure.

Level of education was assessed as the highest level of formal education completed. In Norway, Denmark, and Portugal, the response options ranged from 1 = Primary school (6–8 years at school) to 5 = Higher university level (Master degree, Ph.D. level or similar). In Belgium, additional response options were added to reflect the educational system in the country. To allow for cross-cultural comparisons, this variable was subsequently recoded into 1 = primary (1), 2 = secondary (2 + 3), and 3 = tertiary education (4 + 5).

Religiosity was assessed by asking “Apart from special occasions such as weddings, funerals, and baptisms, how often do you attend religious services or meetings?” Response options ranged from 1 = Once a week or more to 7 = Never.

We used the 12-item Short Form Health Survey (SF12) to assess mental and physical health (Ware, Kosinski, & Keller, 1995). Scoring was done following published guidelines (Ware et al., 1995) to provide overall scores on two sub-dimensions: mental health and physical health. Higher scores indicate better mental and physical health.

Questions on sexual difficulties and distress were adapted from the British Natsal-3 survey (Mitchell et al., 2013). Sexual difficulties were assessed using the following stem “In the last year, have you experienced any of the following for a period of 3 months or longer?” This was followed by eight different sexual difficulties: “lacked interest in having sex,” “lacked enjoyment in sex,” “felt anxious during sex,” “felt physical pain as a result of sex,” “felt no excitement or arousal during sex,” “did not reach a climax (experienced an orgasm)/took a long time to reach a climax despite feeling excited/aroused,” “reached a climax (experienced an orgasm) more quickly than you would have liked,” and “uncomfortably dry vagina.” In the current analyses, we included all but two of the above difficulties; the numbers of women reporting “felt anxious during sex” and “reached a climax more quickly than you would have liked” were too low to permit analyses.

For each difficulty, response options were “yes” or “no”; if a woman responded with a “yes,” she was then asked to indicate how much distress the difficulty had caused her (“no distress,” “mild distress,” “moderate distress,” or “severe distress”). In this article, we use the term “sexual difficulty” to refer to the experience of a sexual difficulty for a period of 3 months or longer in the past year and “distressing sexual difficulty” to denote reporting a sexual difficulty over this time period and also reporting some level of distress (mild, moderate, or severe) about this.

Emotional intimacy was assessed using the 5-item Emotional Intimacy Scale (Sinclair & Dowdy, 2005), which had a high reliability in all four countries (Cronbach’s *α* ranged from .91 to .93). A sample item was “I can share my deepest thoughts and feelings with this person and this person cares deeply for me.” Scores were reverse-coded, so that higher scores indicate higher emotional intimacy.

Sexual intimacy was measured by the following one-item indicator, taken from the validated Natsal-3 SF measure (Mitchell, Ploubidis, Datta, & Wellings, 2012): “I feel emotionally close to my partner when we have sex together.” Responses (1 = always to 5 = hardly ever) were reverse-coded so that higher scores denote higher sexual intimacy.

### Analytic Strategy

We used unweighted data in all analyses presented as the focus was on the women reporting specific sexual difficulties, and not on national averages. While univariate analysis was carried out separately for each country, multivariate OLS regression analysis used a pooled sample controlling for countries (the largest sample, Norwegian women, was employed as the reference group). Robust standard errors were employed. Analyses of predictors of distress were only conducted on the sub-sample that had actually reported that difficulty. The IBM SPSS 24 statistical software package was used for univariate and R version 3.6.0 for multivariate regression analyses.

## Results

Table 1 presents sociodemographic information about participants by country. Age differences among countries were modest, with Norwegian women being the oldest (*M* = 66.5 years, SD= 4.12) and Portuguese women the youngest (*M* = 64.3 years, SD = 3.88). In contrast, significant cross-country differences were found in education levels, particularly between Norwegian and Portuguese women. While percentages of college-educated and only primary school-educated women in the Norwegian sample were 54.1% and 5.6%, respectively, the proportions in the Portuguese sample were 20.7% and 27.1%. Frequency of attending religious services was also markedly different for women across the four countries. Never attending religious services was highest among Belgian (36.9%) and lowest among Danish women (23.7%). Religiosity levels were highest in the Portuguese sample, where 41.6% of participants reported attending religious services at least once a month.

**Table 1:** Sociodemographic characteristics of the sample by country

	Norway *n* (%)	Denmark *n* (%)	Belgium *n* (%)	Portugal *n* (%)	Total *n* (%)
Age (in years)					
60–65	134 (37.5)	103 (32.0)	106 (44.7)	79 (56.0)	422 (39.9)
66–70	125 (35.0)	102 (31.7)	73 (30.8)	47 (33.3)	347 (32.8)
71–75	98 (27.5)	117 (36.3)	58 (24.5)	15 (10.6)	288 (27.2)
Education					
Primary	20 (5.6)	67 (20.8)	26 (11.1)	38 (27.1)	151 (14.4)
Secondary	143 (40.3)	125 (38.8)	123 (52.6)	73 (52.1)	464 (44.1)
Tertiary	192 (54.1)	130 (40.4)	85 (36.3)	29 (20.7)	436 (41.5)
Religiosity					
Never	94 (26.6)	76 (23.7)	86 (36.9)	33 (24.1)	289 (27.7)
Less than once a year	86 (24.3)	83 (25.9)	22 (9.4)	18 (13.1)	209 (20.0)
Once or twice a year	109 (30.8)	116 (36.1)	74 (31.8)	29 (21.2)	328 (31.4)
At least once a month	65 (18.4)	46 (14.3)	51 (21.9)	57 (41.6)	219 (21.0)

Table 2 presents the prevalence of sexual difficulties by country. Across all countries, the most common sexual difficulties reported were orgasmic difficulties, vaginal dryness, and lacking interest in sex. For these three sexual difficulties—orgasmic difficulties, vaginal dryness, and lacking interest in sex—Portuguese women showed the highest prevalence rates.

**Table 2:** Prevalence of sexual difficulties by country

	Norway *n* (%)	Denmark *n* (%)	Belgium *n* (%)	Portugal *n* (%)	Total *n* (%)
Vaginal dryness	138 (38.7)	133 (41.3)	119 (50.2)	74 (52.5)	464 (47.7)
Orgasmic difficulties	159 (51.6)	121 (43.1)	105 (50.0)	78 (63.4)	463 (50.2)
Lacked interest in sex	136 (40.7)	118 (38.8)	140 (62.8)	65 (48.1)	459 (46.1)
Lacked enjoyment	92 (29.5)	76 (26.6)	94 (36.5)	46 (36.5)	308 (33.2)
Pain during sex	64 (20.1)	66 (22.8)	59 (28.2)	33 (26.2)	222 (23.5)
No excitement/arousal	88 (28.7)	71 (25.7)	70 (34.5)	48 (44.0)	277 (30.9)

Next, we analyzed the prevalence of distressing sexual difficulties (i.e., reporting both a sexual difficulty and associated distress by country). About a fourth of participants (26.5%) reported no distressing sexual difficulty lasting 3 months or more in the past 12 months. Among women who reported moderate to severe distress (see Table 3), the most frequent sexual difficulty was vaginal dryness (*n* = 189), followed by problems with reaching orgasm (*n* = 121), and lacking interest in sex (*n* = 116).

**Table 3:** Prevalence of distressing sexual difficulties by country

	Norway		Denmark		Belgium		Portugal		Total	
	No or mild distress	Moderate or severe distress	No or mild distress	Moderate or severe distress	No or mild distress	Moderate or severe distress	No or mild distress	Moderate or severe distress	No or mild distress	Moderate or severe distress
	*n* (%)	*n* (%)	*n* (%)	*n* (%)	*n* (%)	*n* (%)	*n* (%)	*n* (%)	*n* (%)	*n* (%)
Vaginal dryness	59 (48.4)	63 (51.6)	81 (77.1)	24 (22.9)	42 (38.9)	66 (61.1)	24 (40.0)	36 (60.0)	206 (52.2)	189 (47.8)
Orgasmic difficulties	107 (75.9)	34 (34.1)	74 (81.3)	17 (18.7)	59 (59.6)	40 (40.4)	30 (50.0)	30 (50.0)	270 (69.1)	121 (30.9)
Lacked interest in sex	98 (74.2)	34 (25.8)	93 (86.1)	15 (13.9)	82 (65.6)	43 (34.3)	33 (57.9)	24 (42.1)	306 (72.5)	116 (27.5)
Lacked enjoyment	51 (60.7)	33 (39.3)	47 (70.1)	20 (29.9)	51 (58.6)	36 41.4)	20 (52.6)	18 (47.4)	169 (61.2)	107 (38.8)
Pain during sex	27 (43.5)	35 (56.5)	35 (60.3)	23 (39.7)	26 (45.6)	31 (54.4)	9 (40.9)	13 (59.1)	97 (48.7)	199 (51.3)
No excitement/arousal	46 (60.5)	30 (39.5)	42 (77.8)	12 (22.2)	39 (58.2)	28 (41.8)	19 (59.4)	13 (40.6)	229 (63.8)	83 (36.2)

Finally, we explored predictors and correlates of the six distressing sexual difficulties. As presented in Table 4, apart from some between-country differences, we found few significant predictors and correlates. Evidence for differences in the prevalence of sexual difficulties by country was found primarily for orgasmic difficulties and vaginal dryness. Although on average somewhat younger, Portuguese women were characterized by substantially higher frequency of the two most prevalent distressing difficulties (vaginal dryness and problems with orgasm) than Norwegian women. Overall, compared to their Norwegian peers, women in the Danish sample were less likely to report distressing sexual difficulties.

**Table 4:** Predictors and correlates of moderately to severely distressing sexual difficulties in older European women

	Vaginal dryness	Orgasmic difficulties	Lacked interest in sex	Lacked enjoyment in sex	Pain during sex	No excitement or arousal during sex
	*b* (SE)	*b* (SE)	*b* (SE)	*b* (SE)	*b* (SE)	*b* (SE)
Denmark	− 0.33 (.11)**	− 0.24 (.11)*	− 0.25 (.12)*	− 0.07 (− 16)	− 0.15 (.17)	− 0.50 (.17)**
Belgium	0.24 (.13)	0.27 (.13)*	0.15 (.14)	0.21 (.16)	− 0.11 (.20)	− 0.08 (.17)
Portugal	0.37 (.16)*	0.32 (.15)*	0.18 (.18)	0.21 (.20)	− 0.06 (.27)	− 0.16 (.22)
Age	− 0.00 (.01)	0.01 (.01)	− 0.02 (.01)	− 0.00 (.02)	0.00 (.02)	− 0.00 (.01)
Secondary education	0.19 (.14)	− 0.01 (.15)	− 0.11 (.18)	− 0.02 (.21)	0.22 (.28)	− 0.09 (.21)
Tertiary education	0.38 (.15)*	0.20 (.15)	0.01 (.19)	0.34 (.21)	0.42 (.28)	0.08 (.20)
Religiosity	− 0.04 (.02)	0.03 (.02)	0.03 (.03)	0.01 (.03)	− 0.02 (.04)	0.01 (.03)
Sexual intimacy	0.05 (.07)	− 0.15 (.06)*	− 0.03 (.06)	− 0.02 (.07)	− 0.01 (.10)	− 0.12 (.08)
Emotional intimacy	− 0.02 (.01)*	− 0.01 (.01)	− 0.00 (.01)	− 0.01 (.02)	− 0.02 (.01)	− 0.00 (.01)
Mental health (SF-12)	0.01 (.01)	− 0.01 (.01)	− 0.01 (.01)	− 0.02 (.01)*	− 0.01 (.01)	− 0.00 (.01)
Physical health (SF-12)	− 0.00 (.00)	− 0.00 (.01)	− 0.00 (.01)	− 0.00 (.01)	0.00 (.01)	0.00 (.01)
Number of sexual difficulties	0.19 (.03)***	0.19 (.03)***	0.16 (.03)***	0.20 (.04)***	0.14 (.06)*	0.23 (.05)***
*R*^2^	.26	.30	.19	.22	.17	.22

*p < .05, **p < .01, ***p < .001

College or university education was related to distressing vaginal dryness (*b* = 0.38, *p* = .014); compared to women with only primary education, highly educated women were more distressed about this symptom. No other significant sociodemographic predictors were observed across the remaining sexual difficulties.

Emotional intimacy was significantly and negatively related to distress about vaginal dryness (*b* = − 0.02, *p* = .022); the higher the reported emotional intimacy with a partner, the lower the distress. Similarly, sexual intimacy was significantly associated with distressing orgasmic difficulties (*b* = − 0.18, *p* = .020), with higher levels of closeness with a partner during sex associated with lower distress about orgasmic function. Finally, mental health status was related to distress about lack of enjoyment in sexual activities (*b* = − 0.02, *p* = .018); women with better mental health scores reported less distress about not enjoying sex.

The proportion of explained variance for the six distressing sexual difficulties was modest, ranging from 17% (for pain during sex) to 30% (for orgasmic difficulties).

## Discussion

In this study of women aged 60–75 years, we found a fairly high prevalence of sexual difficulties lasting 3 months or longer in the past year across the four European countries assessed. The most common problems were difficulties with vaginal dryness, reaching orgasm, and lacking interest in sex, with prevalence rates ranging between 37 and 59%. Consistent with previous research (Lee et al., 2016; Mitchell et al., 2016), however, the majority of women who reported sexual difficulties reported no or mild distress about these difficulties.

It is difficult to compare our prevalence estimates with those of most previous studies because of varied definitions of sexual difficulties, survey questions, age of sample, etc. We did use the same questions as in the Natsal-3 survey (Mitchell et al., 2013) to assess sexual difficulties and associated distress, although the age categories of participants were somewhat different (60–75 years in our survey, compared with 55–64 and 65–74 years in Natsal-3). The most common three sexual problems reported both in our study and in the Natsal-3 survey were vaginal dryness, difficulties reaching orgasm, and lacking interest in sex. Reports of these three difficulties, however, were considerably higher in our sample compared with the Natsal-3 survey (vaginal dryness 47.7% vs. 20.0%; difficulties reaching orgasm 50.2% vs. 13.7%; lacking interest in sex 46.1% vs. 34.2%). In the analysis on older men in our survey, the percentages reporting sexual problems were also substantially higher than in the Natsal-3 survey (Hald et al., 2019). One possible reason for the discrepant rates in the two surveys might be the fact we used postal questionnaires, whereas in Natsal-3, a combination of computer-assisted face-to-face interviews and self-completion questionnaires was used.

Few previous studies have investigated predictors/correlates of distress about specific sexual difficulties in older women; instead, most research has focused on predictors of sexual problems (with or without accompanying distress). We investigated age, education, and religiosity as possible predictors of distress about sexual difficulties and found only one significant predictor: compared to women with only primary education, college-educated women reported more distress about vaginal dryness. It is possible that sex education (not assessed in the current study) rather than general educational level might be associated with the likelihood of reporting distressing sexual difficulties. Using Natsal-3 data, Macdowall et al. (2015) found that women citing school as their main source of sex education relative to other sources (e.g., parents) reported less distress about sex over the past year.

Although the early literature on the successful aging model included social, mental/emotional, and physical aspects of well-being (Woloski-Wruble et al., 2010), researchers have recently explored the role that sexuality plays in successful aging (Štulhofer, Hinchliff, Jurin, Carvalheira, & Træen, 2019; Thompson et al., 2011). Our findings are consistent with this expanded model of successful aging and underline the close links between sexual and nonsexual aspects of older women’s lives and the importance of sexuality for general life satisfaction (Woloski-Wruble et al., 2010). Factors that were associated with distress about specific sexual difficulties were emotional intimacy (negatively related to distressing vaginal dryness), sexual intimacy (negatively related to distress about orgasm difficulties), and mental health (better mental health negatively related to distress about lacking enjoyment in sex). It is interesting that physical health did not feature as a predictor of distress for any of the sexual difficulties assessed. In contrast, Hald et al. (2019) reporting on older men from the same sample as in our study, found poor physical health was a significant predictor of overall distress about sexual difficulties.

Our findings related to emotional and sexual intimacy are consistent with a study of U.S. women aged 20–65 years, where the best predictors of sexual distress were markers of emotional well-being and emotional intimacy with the partner during sexual activity (Bancroft et al., 2003). Other studies have highlighted the central role of emotional intimacy in aging women and men (Fileborn et al., 2017; Heiman et al., 2011; Müller et al., 2014). Qualitative studies provide some insight into how emotional intimacy might be a protective factor for older women’s and men’s sexuality. For example, Erens et al. (2019) found that among older women and men who reported having a health condition or taking medication that affected their sex life, those in close relationships described compensatory mechanisms to deal with sexual problems and seemed less distressed than those in relationships that were less close. In interviews with men aged 60 and older, Fileborn et al. observed that men frequently talked about the centrality of intimacy and bonding in their sexual relationships.

Overall, we found relatively few cross-country differences in both the prevalence of sexual difficulties and distressing sexual difficulties. This is interesting, given that there were marked cross-country differences in education and religiosity and expected differences in gender roles/gender inequality. It seems, therefore, that sociodemographic factors may not exert a strong influence on the prevalence of sexual difficulties in older women, although other sociodemographic factors, such as income or social support, may have a greater impact on whether or not a woman reports sexual difficulties and/or distress about these difficulties.

### Strengths and Limitations

Strengths of our study include the large sample size and the inclusion of women from four European countries. There were also several limitations that need to be acknowledged. Firstly, we only included women who reported being sexually active in the last year and in committed relationships and thus, our sample is not representative of older women in general. As older women who have sexual difficulties may avoid sexual activity and relationships (Carvalheira, Štulhofer, Graham, & Træen, 2019), we may have under-estimated the prevalence of some sexual difficulties. The meaning of moderate/severe distress might also be different between sexually active and sexually inactive older women, which could in turn affect the associations between distressing sexual difficulties and predictors. Secondly, we recruited women of various sexual orientations, but because of small numbers of women who reported any sexual orientation other than “heterosexual” (only 38 women identified as gay or lesbian, bisexual, or other), we could not analyze possible differences based on sexual orientation. Thirdly, the response rate in the Portuguese sample was very low, which raises questions about its comparability with other samples. Because of our recruitment method (simple random sampling from national phone registries) and reasonably low refusal rates (particularly in Norway), we are confident that the Norway, Denmark, and Belgian samples reflect the older populations in those countries well. In Portugal, however, because of the difficulty recruiting, higher educated and urban individuals were oversampled. Fourth, the cross-sectional design of the study precludes making any conclusions about causal relationships. Fifth, the questionnaire was developed in English and subsequently translated using the translation and back translation method into local languages by the principal investigators and persons employed by IPSOS in each country. It is possible that the some of the questions on the translated versions of the questionnaire might have been understood differently by respondents in different countries.

Finally, the proportion of explained variance in the six distressing sexual difficulties was modest (17–30%, depending on specific sexual difficulty), which points to the existence of other, unmeasured sociodemographic, relational, and psychological factors that are relevant for understanding sexual distress in older women. Perceived importance of sex is one likely relevant variable. In a sample of older women and men from the National, Social Life, Health, and Aging Project, those who viewed sex as important were more likely to be sexually distressed than individuals who rated sex as less important (Juang & Knight, 2018). In a study of younger women (M age, 27.4 years), Stephenson and Meston (2015) found that sexual problems were distressing primarily because of the reduction in pleasure experienced either by women themselves or by their partners. Future studies should include these potential predictors in addition to those found important in this survey. Qualitative studies of older women’s experiences of distressing sexual difficulties should endeavor to identify other possible predictors—including the perspective of the partner—to shed light on the possible mechanisms that might explain why some women experience distress about sexual problems, while others do not.

### Clinical and Research Implications

This study underlines the importance of assessing distress about sexual difficulties among older women, both in research studies and in clinical contexts. Clinicians should not necessarily assume that age-related sexual difficulties are associated with concern or distress, nor with the absence of sexual activity among their older female patients. Considering that the proportion of older adults who seek help for sexual problems appears to be very low (Hinchliff & Gott, 2011), clinicians should ask about and carefully assess sexual difficulties and distress and determine whether their older adult female patients would like to receive professional help for their sexual difficulties. Moreover, clinicians should be aware of their own attitudes toward the sexuality of older adults.

### Conclusion

The findings indicate that many older women report sexual difficulties, but many also experience only mild or no distress about these difficulties. Few cross-country differences in the prevalence of sexual difficulties or of distressing sexual difficulties were evident. Levels of emotional and sexual intimacy during sex, and mental health, predicted whether women reported distress about specific sexual difficulties. The findings underline the importance of clinicians asking older women about sexual function and associated distress. More and careful attention to the psychological and relationship context of these sexual difficulties and its related distress is needed, as these could be important targets in the treatment process of older women consulting for distressing sexual difficulties.
